# Supplementary material for: Reduced and unstratified crust in CV chondrite parent body
Source: Nat Commun. 2017 Aug 15;8:261. doi: 10.1038/s41467-017-00293-1 (PMC5557907; doi:10.1038/s41467-017-00293-1)
Supplement: Supplementary file 1 — Supplementary Information [file 41467_2017_293_MOESM1_ESM.pdf]

File name: Supplementary Information
Description: Supplementary Figures

File name: Peer Review File
Description:

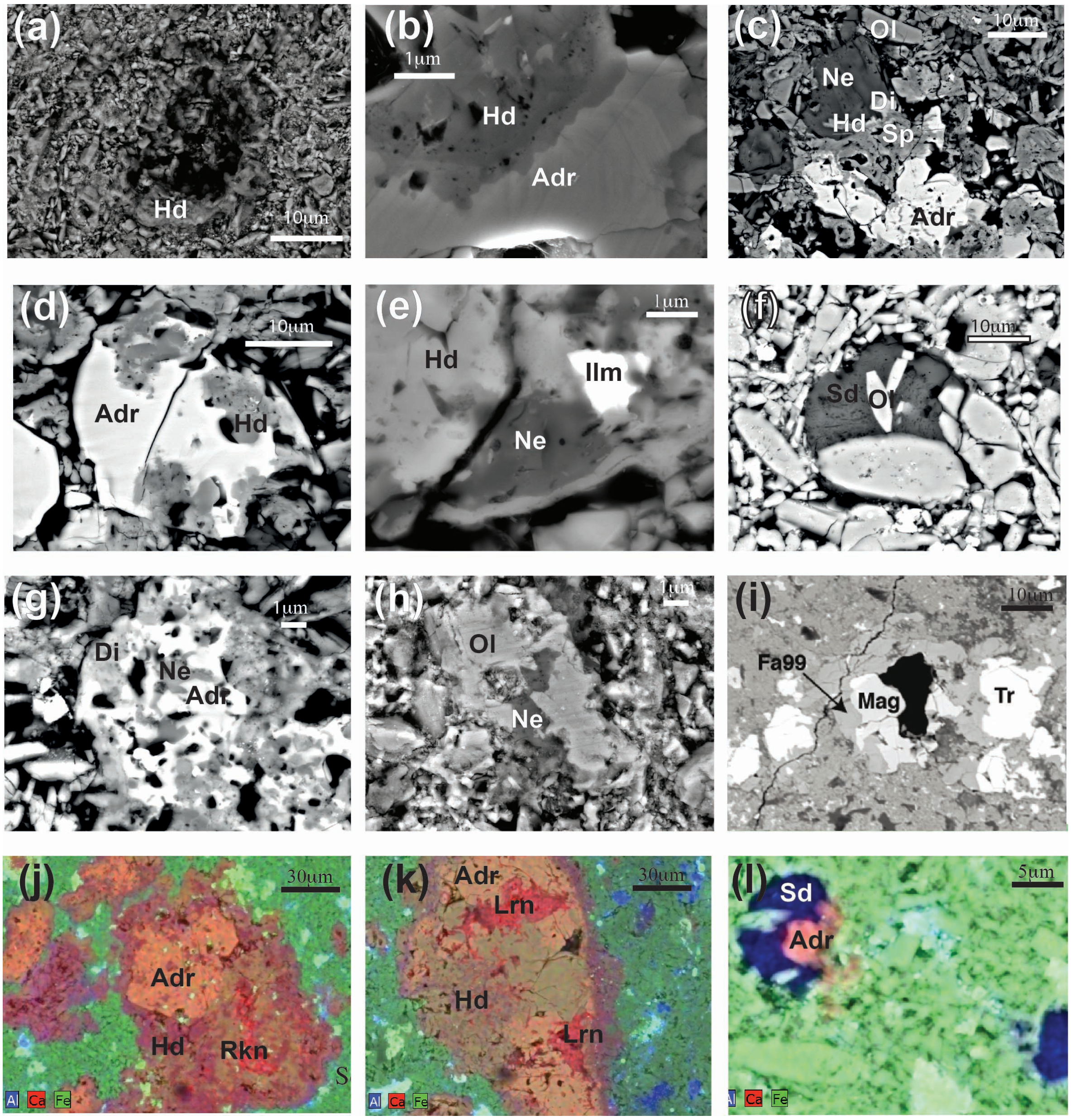

Supplementary Fig. 1: Details of secondary minerals assemblages in CV chondrites. Backscattered Electron images of the matrix of Allende (a-h), and Vigarano (i). Backscattered Electron images coupled with Energy-Dispersive X-ray chemical maps (red=calcium; blue=aluminum; green=iron) of Bali (j-l). (a) diopside-crusted cavity; (b) zoned andradite associated to hedenbergite; (c) Andradite (Adr), Spinel (Sp), Hedenbergite (Hd), Nepheline (Ne), Fayalite 50 (Fa) association; (d) andradite – hedenbergite inclusion relationship; (e) ilmenite (Ilm) associated with Hd and Ne ; (f) fayalite 50 included in sodalite (Sd); (g) Nepheline and diopside enclosed in Andradite relict; (h) Nepheline filling fracture in Olivine (Fa50); (i) Fa-Mt-Sulfide (troilite) association; (j) rankinite associated with andradite surrounded by hedenbegite (k) larnite enclosed in andradite; (l) andradite in a sodalite filled cavity.

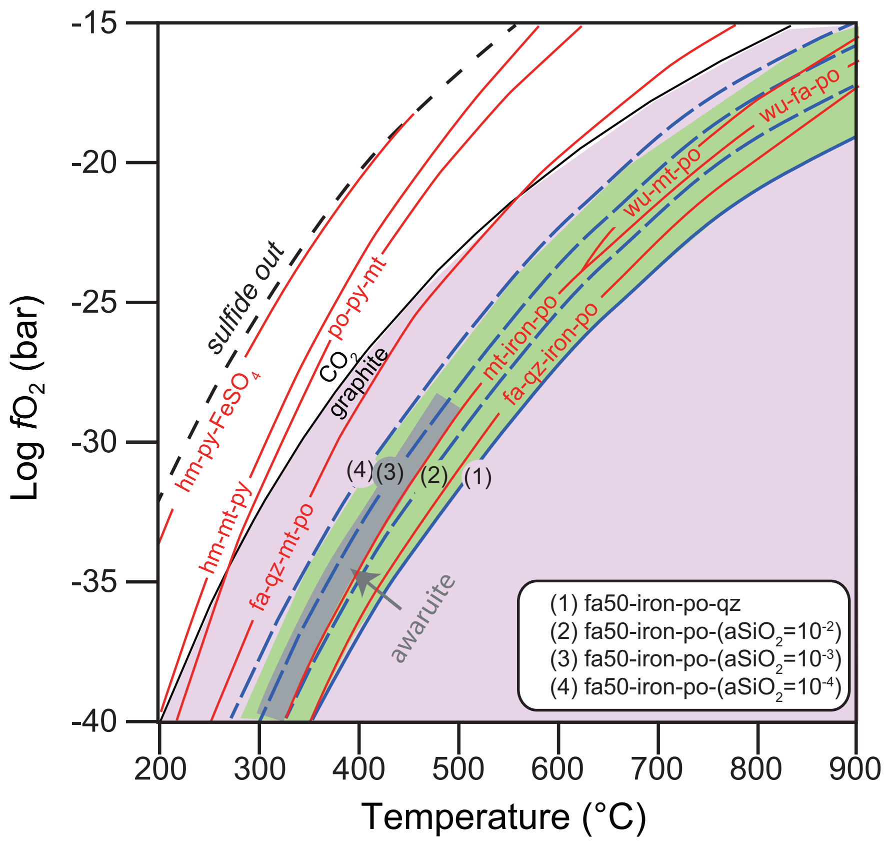

Supplementary Fig. 2: Oxygen fugacity versus temperature plot of the stability fields of typical phases in CV chondrites matrices. The presence of Fe sulfide ± fayalite40-60 ± magnetite ± Fe,Ni metal ± awaruite ± graphite is in good agreement with intrinsic oxygen fugacity around IM-buffered conditions.
